# Supplementary material for: Containment of a healthcare-associated COVID-19 outbreak in a university hospital in Seoul, Korea: A single-center experience
Source: PLoS One. 2020 Aug 14;15(8):e0237692. doi: 10.1371/journal.pone.0237692 (PMC7428087; doi:10.1371/journal.pone.0237692)
Supplement: Attachment — Submitted filename: Response to Reviewers_20200711.docx [file pone.0237692.s001.docx]

Title: Containment of a healthcare-associated COVID-19 outbreak in a university hospital in Seoul, Korea: a single-center experience

Authors: Sei Won Kim, Sung Jin Jo, Heayon Lee, Jung Hwan Oh, Jihyang Lim, Sang Haak Lee, Jung Hyun Choi, Jehoon Lee

Dear Dr. Jin

We really appreciate that the reviewer has provided valuable comments on how to improve our research. We fully understood the points from the reviewer and revised the manuscript.

**< Reviewer #1 >**

1. This paper is not written clearly. The procedures taken is not easy to follow and confusing. For example, page 16 “14 healthcare-associated COVID-19 cases were reported in our hospital.” This number looks like including subsequent confirmed cases related to the hospital illustrated in Figure 2. Without clean explanation this sentence is confusing.

: Thank you very much for the comment. Fourteen healthcare-associated COVID-19 cases consisted of four in-hospital diagnoses and ten out-of-hospital diagnoses. For clear explanation, we revised the figures and manuscript.

2. This paper conclude hospital shutdown for extended period was not necessary and testing of all hospital staff and inpatients without risk assessment are time-consuming. However, the paper does not provide enough scientific evidence how this conclusion is reached. Low confirmed probability ex-post is not the evidence you can use since the risk of outbreak is still exist. Just explaining the action taken by Korea is meaningful.

: We fully agree with the comment and revised the manuscript. The revised conclusion is as follows. “In conclusion, the robust control of the COVID-19 outbreak further minimized the transmission of SARS-CoV-2 in the hospital and local communities. However, there was also a debate over the appropriate period of hospital shutdown and testing of all hospital staff and patients. Future studies are required to refine and establish the in-hospital quarantine and de-isolation guidelines based on the epidemiological and clinical settings.”
